# Importance of Using Angiography for the Early Detection of Chronic Limb Ischemia in Diabetic Foot Wounds

**DOI:** 10.7759/cureus.61906

**Published:** 2024-06-07

**Authors:** Vivie Tran, Bernardo Galvan, Sachi Khemka, Katherine Holder, Mohammad M Ansari

**Affiliations:** 1 Internal Medicine, Texas Tech University Health Sciences Center, Lubbock, USA; 2 General Surgery, Texas Tech University Health Sciences Center, Lubbock, USA; 3 Dermatology, University of Iowa Health Care, Iowa City, USA; 4 Cardiology, Texas Tech University, Lubbock, USA; 5 Cardiology, Texas Tech University Health Sciences Center, Lubbock, USA

**Keywords:** diabetes mellitus, early diagnosis and treatment, tailored interventions, revascularization interventions, peripheral artery disease (pad)

## Abstract

Peripheral artery disease (PAD) affects millions of people worldwide, presenting with varying symptom severity, including chronic total occlusion of arteries, and occasionally, limb amputation. There are various interventions, such as atherectomy and the use of drug-coated balloons and stents, which have been developed to revascularize affected ischemic regions. However, each interventional approach must be individualized due to a patient’s unique underlying conditions. Comorbid conditions, especially diabetes, play a significant role in PAD, as poorly controlled diabetes can accelerate PAD progression. For this reason, an early and accurate diagnosis of PAD is crucial, especially when symptoms may present dissimilar to classic PAD symptoms, often leading to misdiagnosis. The presented cases highlight the tailored interventions to revascularize arteries in patients with diabetic foot wounds utilizing catheters, stents, guidewires, and balloons, made possible after early angiogram. These interventions have been promising in treating PAD patients, and highlight the need for early diagnosis and timely and customized interventions to prevent limb amputation and mitigate potential complications.

## Introduction

Peripheral artery disease (PAD) affects over 200 million people worldwide, causing asymptomatic disease in some while resulting in devastating pain and disability in others [1]. Chronic total occlusion (CTO) refers to 100% occlusion of an artery for three or more months, which may result in severe ischemic, claudication, and necrosis in PAD patients [2].

Amputation becomes necessary in 3-4% of PAD patients, making the disease a leading cause of limb loss in the developed world. A plethora of procedures and devices, including angioplasty, drug-coated balloons, and stents, have been developed and refined over the past two decades to mitigate the adverse events associated with chronic limb ischemia [3]. Data in support of intervention are becoming increasingly robust, but the most efficacious intervention methods are still highly debated with a variety of strategies and techniques currently in use [4]. In the case of CTO of the peripheral vasculature, femoral or popliteal access is commonly used to access the diseased vasculature, as the larger vessels are considered convenient to access [5]. While further innovation is needed for PAD treatment, new evidence suggests that a correct and early diagnosis of PAD may be equally important in improving outcomes for PAD patients [6-8].

PAD is closely tied to comorbid conditions, including diabetes [9]. Diabetics often present with integumentary manifestations that mimic PAD symptoms. Diabetic patients with poor glucose control demonstrate rapid acceleration of PAD and its associated symptoms [10]. A recent study demonstrated that in diabetic patients who are screened with an Ankle-Brachial Index (ABI) test, the presence of PAD is about 13% [11]. Additionally, diabetes increases the prevalence of symptomatic PAD by 3.5x in men and 8.6x in women [9]. Gangrenous and ischemic lower limb tissue in diabetic patients is often attributed solely to their diabetes [12]. This may be especially true in smaller centers or for patients with uncontrolled diabetics and contributes to misdiagnosis and undertreatment of PAD in diabetic patients [13].

Recent multicenter cohort studies have shown that at least half of diabetic foot ulcers are of ischemic origin [14,15]. Diagnosing CTO in patients with diabetes is especially challenging, as less than 25% of diabetic patients with PAD report intermittent claudication. Additionally, diabetic neuropathy reduces symptoms of rest pain in this patient population. The altered symptomatic presentation of PAD in diabetic patients delays correct diagnosis and treatment. In diabetic patients, 30-50% of foot ulcers are already gangrenous by the time vascular consultation is arranged, hindering effective intervention [16]. This case series delves into a special technique involving the sequential combined utilization of multiple catheters, guidewires, balloons, and stents to effectively treat advanced peripheral artery disease in a diabetic patient. The feasibility of this technique emerged after an ultrasound examination, which proved insufficient in depicting the intricate blood flow in the small vessels entering the foot. However, upon conducting an angiogram, the presence of vascular stenosis became distinctly evident, enabling us to successfully address the issue and aid the patient. In essence, the success of this endeavor was made possible through the guidance provided by the angiogram.

## Case presentation

A male, aged 77, with a history of critical limb ischemia (Rutherford VI) presented for evaluation of a gangrenous wound that failed to heal. The patient has a past medical history of coronary artery disease (CAD), distal abdominal aortic aneurysm, diabetes mellitus (DM) type II, and hypertension (HTN). He presented initially with a gangrenous right first and second toe that had progressively worsened over the past two months but was initially attributed primarily to the patient’s diabetes. Treatment with antibiotic ointment and wound care provided no improvement. A peripheral angiogram was performed and revealed a severe extent of occlusion throughout the left lower limb, prompting the patient to be emergently taken to the catheter lab for reperfusion via angioplasty and stenting. Using a special, tailored technique, the left femoral artery was used to gain access to the occluded vasculature via an ultrasound guide probe, and the wire was advanced down the superficial artery crossing into the right anterior tibial with the help of a crossing catheter. After crossing, balloon angioplasty was successfully performed. Arthrectomy was also performed due to an occluded, calcified right superficial femoral artery. A series of balloons were advanced and inflated in the superficial femoral, anterior, and posterior tibial arteries. Next, balloons were carefully exchanged and deployed in the right dorsalis pedis and distal anterior tibial arteries, and angioplasty was performed. Self-expanding coated stents were subsequently placed in the right superficial femoral artery. Drug-coated balloons were deployed proximal to the stent to prevent stent restenosis. What was initially thought to be a non-healing diabetic wound was correctly diagnosed as an extensive CTO of the patient’s lower limb. The post-procedure course was unremarkable, and the patient was discharged the day after the procedure with instructions for follow-up care.

**Figure 1:**
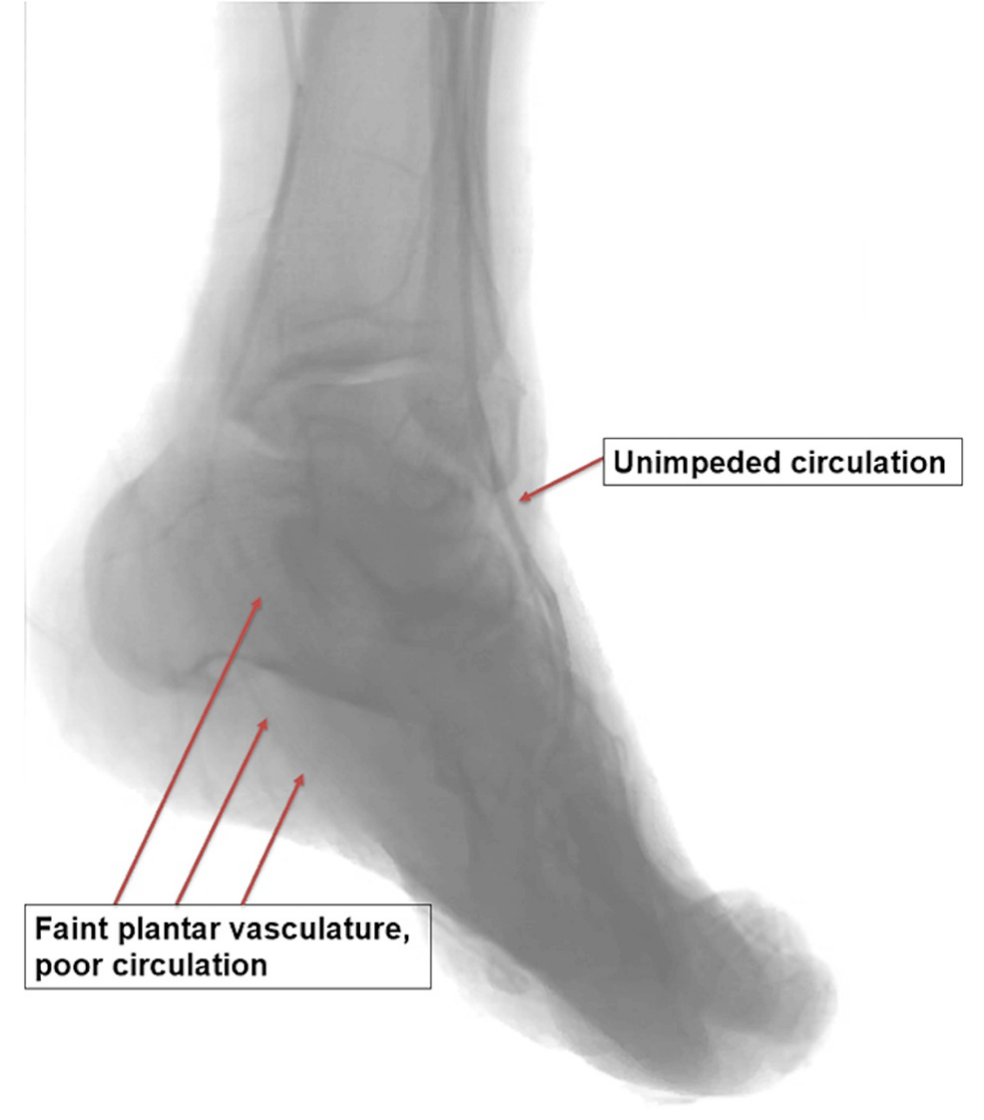
Pre-atherectomy plantar occlusion

**Figure 2:**
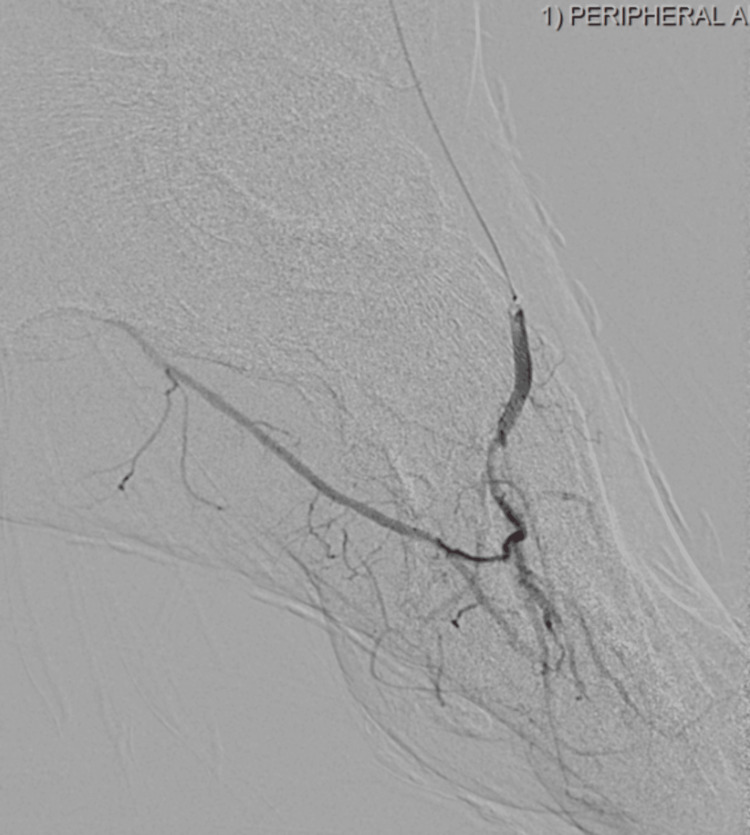
Post-atherectomy revascularization

**Figure 3:**
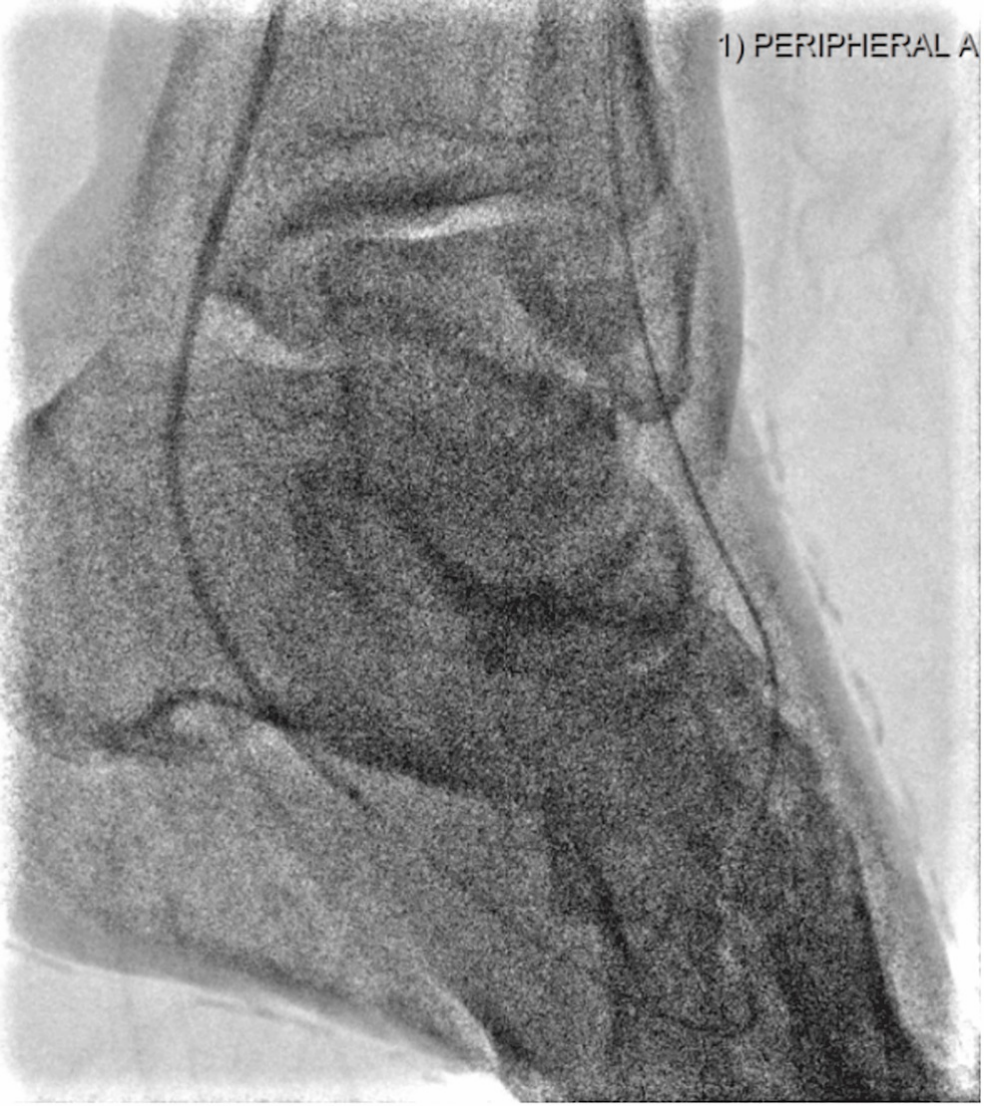
Post-atherectomy unobstructed plantar vessels

A male, aged 35, with a history of critical limb ischemia (Rutherford IV) presented with bilateral foot ulcers that were not healing and had worsened over a two-month period. The patient had a medical history of PAD, osteomyelitis in the right lower extremity (RLE), HTN, dyslipidemia (DLD), critical limb ischemia (CLI), and DM type II. The patient had previously undergone partial amputation of the right third digit, but bilateral swelling continued due to his diabetes. Despite wound care and intravenous antibiotics, bilateral swelling persisted. A peripheral angiogram of both lower extremities revealed partial occlusion in the left lower limb and complete occlusion of the left plantar arch (Figure 1). The procedure involved replacing the femoral sheath with a 6-Fr, 45 cm long destination sheath positioned at the left common femoral artery. Initial attempts to cross the occlusion in the medial plantar artery were unsuccessful, but the repositioning of the wire into the anterior tibial artery allowed progress. Subsequently, a FineCross catheter (Terumo Interventional Systems, Somerset, NJ, USA) was advanced, enabling angiography of the left foot, with further advancement into the dorsalis pedis artery. To address the occlusion, a Gladius wire (Asahi Intecc USA, Inc., Irvine, CA, USA) was exchanged and advanced into the plantar arch, facilitating retrograde crossing with the support of the FineCross catheter. Balloon angioplasty was performed using a Crostella balloon (Terumo Interventional Systems) in the medial plantar artery, followed by the plantar arch, dorsalis pedis, and distal anterior tibial artery. Additional interventions included the use of a Coyote balloon (Boston Scientific, Marlborough, MA, USA) in the proximal anterior tibial artery, tibioperoneal trunk (TPT), and peroneal artery. A drug-coated balloon (DCB) was utilized in the distal popliteal artery. Post-intervention angiography confirmed successful revascularization without complications, and the patient was discharged from the catheterization laboratory in a stable condition with an intact pedal pulse (Figures 2, 3).

A male, aged 56, with a history of PAD, osteomyelitis, hypothyroidism, HTN, and end-stage renal disease presented to the clinic with a wet gangrenous diabetic ulcer of the left foot and underwent ray amputation of the left second, third, and fourth toes. Treatment with antibiotics and subsequent wound care resulted in no significant improvement. Peripheral angiography revealed significant stenosis and occlusion along the left lower limb. The procedure involved advancing a glide wire to the left iliac artery using a hook catheter. The femoral sheath was then replaced with a 6-Fr and 45 cm destination sheath placed at the left common femoral artery. A Regalia wire ((Asahi Intecc USA, Inc.) was guided to the posterior tibial artery with the assistance of a Navicross catheter (Terumo Interventional Systems) and placed in the distal posterior tibial artery using a FineCross catheter. The wire was then replaced with a Straub wire (Straub Medical AG, Vilters-Wangs, Switzerland) using a TrailBlazer catheter (Medtronic plc, Dublin, Ireland). Rotational atherectomy was performed in the superficial femoral artery (SFA), popliteal artery, and TPT for two passes. Balloon angioplasty was subsequently performed at the distal posterior tibial, proximal TPT, and popliteal and distal common femoral artery (CFA) using a Crostella balloon. The Ultraverse balloon (BD, Franklin Lakes, NJ, USA) was used to inflate proximally at the CFA and proximal SFA. Further attempts were made to access the distal medial plantar artery using different wires but were unsuccessful until a Regalia wire was advanced successfully. Additional balloon angioplasty was performed in the anterior tibial artery and dorsalis pedis artery. Drug-coated balloons (Lutonix DCB; BD) were used in the distal SFA and CFA to improve the outcome. The post-procedure angiography revealed excellent outcomes without any complications. The patient was stable, with preserved blood flow to the affected area, and palpable pulses of the left dorsalis pedis and posterior tibial arteries.

## Discussion

Intervention in chronic total occlusion of the peripheral vasculature requires specialized techniques that may be optimally effective when tailored to each patient. This becomes increasingly important in patients with peripheral artery disease who also have diabetes as a primary comorbidity.

Diabetic foot wounds are customarily managed with wound care and antibiotics [17]. Prevention and treatment of diabetic foot wounds have traditionally been focused on neuropathy and its consequences, although ischemia is the most important factor preventing healing [18]. Both macrovascular disease and microvascular dysfunction secondary to collagen glycation impair perfusion in the lower limbs of diabetic patients [19].

Additionally, peripheral autonomic neuropathy causes altered blood flow regulation to the lower extremities, opening arteriovenous shunts and causing precapillary sphincter malfunction [9]. This contributes to the appearance of warm, dry skin and increases the likelihood of wound formation. Subsequent capillary leak, venous pooling, and inflammation also contribute to decreased perfusion in the diabetic foot. This indicates that decreased perfusion in the lower extremities of diabetic patients is complex, but PAD is the most important factor [20].

PAD in patients with diabetes is often multisegmental and infrapopliteal and presents with few collateral vessels [20]. Ischemia is reported to be a contributing factor in at least 90% of diabetic patients undergoing amputation [9].

In the wound clinic, a significant number of diabetic patients present with foot wounds, commonly labeled as diabetic foot wounds. However, the prevailing treatment approach often focuses solely on superficial wound care. It's crucial to recognize the multifaceted nature of these wounds, which often coexist with underlying vascular disease. While ultrasound and ABI tests are routinely conducted, ultrasound may fall short in accurately detecting slow-flowing and small foot vessels. Consequently, vascular experts now advocate for the use of angiograms, which have emerged as a more definitive diagnostic tool. Complementing this, CT angiograms are increasingly recommended to effectively rule out peripheral vascular disease. Once a diagnosis is established, addressing the vascular component of the disease becomes paramount, leading to enhanced wound-healing outcomes in diabetic patients.

## Conclusions

Lower extremity circulation is often not thoroughly investigated, and in cases of peripheral artery disease, reduced blood flow due to critical limb ischemia hampers the essential healing process. While the specific treatment approach is commendable in this instance, what's perhaps more intriguing is the possibility that vascular issues in this patient may have been mistakenly attributed solely to diabetes, without a high level of clinical suspicion and meticulous examination.

Following two months of unsuccessful wound care, an angiogram was eventually performed, revealing the presence of critical limb ischemia. With a correct diagnosis in hand, a tailored strategy was implemented, involving peripheral transluminal angioplasty to clear the blocked vessels, resulting in positive outcomes that prevented further lower limb ischemia complications. This pattern was also observed in two other cases and several patients with similar underlying causes. Our case series highlights the necessity of a comprehensive approach to diabetic wound healing, emphasizing the importance of recognizing the limitations of ultrasound and the imperative to assess or diagnose vascular disease in diabetic patients through peripheral angiography. This strategic approach significantly contributes to expediting the wound-healing process post-operation.
